# Impact of sidedness of colorectal cancer on tumor immunity

**DOI:** 10.1371/journal.pone.0240408

**Published:** 2020-10-12

**Authors:** Chie Takasu, Masaaki Nishi, Kozo Yoshikawa, Takuya Tokunaga, Hideya Kashihara, Toshiaki Yoshimoto, Mitsuo Shimada

**Affiliations:** Department of Surgery, The University of Tokushima, Tokushima, Japan; Howard University, UNITED STATES

## Abstract

**Background:**

Clinical and molecular characteristics differ between right-sided and left-sided colorectal cancer (CRC). This study aimed to clarify the correlation between CRC sidedness and tumor immunity.

**Methods:**

A total of 102 patients who underwent curative colectomy for stage II/III CRC were included in this study. The expression of programmed cell death (PD)-1, PD1-ligand 1 (PD-L1), forkhead box P3 (Foxp3), transforming growth factor (TGF)-β, and indoleamine-pyrrole 2,3-dioxygenase (IDO) were examined using immunohistochemistry and the relationships between sidedness and several prognostic factors were examined.

**Results:**

Clinicopathological factors were not significantly different between right- and left-sided CRC. The tumor immunity-related molecule PD-L1 was more highly expressed in right-sided than in left-sided CRC (62.9% vs. 30.6%, p<0.01). No significant difference was found in overall survival (OS) and disease-free survival (DFS) by sidedness. PD-1 and Foxp3 expression were significant prognostic factors for OS. Lymph node metastasis (N), lymphatic invasion (ly), and PD-L1 expression were significant prognostic factors for DFS. In right-sided CRC, IDO-positive patients had a poor OS (p<0.05), and IDO was the only independent prognostic indicator for OS. N and venous invasion were identified as independent prognostic indicators for DFS. In left-sided CRC, univariate analysis identified PD-1, PD-L1, and Foxp3 expression as significant predictors of poor OS. Multivariate analysis confirmed PD-L1 expression as an independent prognostic indicator. N, ly, and PD-L1 expression levels were identified as significant predictors of poor DFS.

**Conclusions:**

The prognostic factors were IDO in right-sided CRC and PD-L1 and Foxp3 in left-sided CRC. These findings indicated that tumor immunity might play different roles depending upon sidedness. Tumor location may be an important factor to consider when assessing immune response and therapeutic decisions in CRC patients.

## Introduction

Colorectal cancer (CRC) is one of the most common cancers in both men and women worldwide. Research in CRC has been focused on providing more personalized treatment based on genetic and molecular mechanisms of carcinogenesis. Accumulating evidence indicates that CRC shows differences in pathogenesis, molecular pathways, and prognosis depending on the sidedness [1].

Chromosomal instability is associated with 60%-70% of CRC and frequently observed in left-sided CRC. Furthermore, Kirsten rat sarcoma viral oncogene homolog (KRAS) and p53 mutations have been characterized as left-sided CRC [2]. In contrast, microsatellite instability (MSI)-high, B-Raf proto-oncogene, serine/threonine kinase (BRAF) mutation, and CpG island methylator phenotype (CIMP)-high are often characterized as right-sided CRC [1, 3]. The seminal phase II trial reported that metastatic CRC with deficient DNA mismatch repair (dMMR) and MSI-high responds to programmed cell death 1 (PD-1) inhibitors while CRCs with proficient MMR and microsatellite stable (MSS) do not [4]. We previously reported that PD-1 and PD-1 ligand 1 (PD-L1) expression levels were associated with poor prognosis and correlated with transforming growth factor (TGF)-β and forkhead box P3 (Foxp3) expression in CRC patients [5]. Previous report revealed reported immune cell-specific PD-L1 and PD-1 expression as prognostic factors depending on the sidedness [6]. However, the characteristics of tumor immunity have not been investigated in relation to the tumor sidedness. Therefore, the aim of this study was to reveal the correlation of CRC sidedness with tumor immunity.

## Methods

### Patients

For 5 years from April 2004 to April 2009, 102 patients who underwent curative colectomy for stage II/III CRC in the Tokushima University Hospital were included in this study. Tumors located in the cecum and ascending and transverse colon up to the splenic flexure were defined as right sided, whereas those of the descending colon, sigmoid colon, and rectum were defined as left sided [1].

There were 69 men and 34 women, with a mean age of 68.7 (range, 41–90) years. The mean follow-up period were 60 (range 11–158) months. Fifty-two patients underwent adjuvant chemotherapy. Factors were defined according to the 8th edition of the Japanese Classification of Colorectal Carcinoma. Each participant provided written, informed consent prior to participation in this study, and this study was authorized in advance by the institutional review board of the University of Tokushima Graduate School of Medical Science (Approved #2347).

### Immunohistochemistry

Tissue samples were formalin fixed, paraffin embedded, cut into 5 μm-thick serial sections, which were dewaxed, deparaffinized in xylene, and rehydrated using a series of graded alcohol concentrations. Samples were boiled for 20 min in a microwave oven in citrate buffer (pH 6.0) for antigen retrieval. Endogenous peroxidases were blocked with 0.3% hydrogen peroxide for 30 min, followed by incubation in 5% goat serum for 60 min to prevent nonspecific antigen binding. The slides were then incubated with primary antibodies overnight at 4°C. The following primary antibodies and dilutions were used: mouse monoclonal antibody against PD-1 (AF1086, 1:40; R&D Systems, Minneapolis, MN, USA), rabbit monoclonal antibody against PD-L1 (ab174838, 1:100; Abcam, Cambridge, UK), and mouse monoclonal antibodies against Foxp3 (ab20034, 1:100; Abcam), IDO (ab71276, 1:50; Abcam), and TGF-β (sc-146, 1:100; Santa Cruz). Secondary antibody binding was detected using Histofine SAB-PO (Nichirei) for PD-1 and EnVision Dual Link System-horseradish peroxidase (HRP, DAKO) for PD-L1, Foxp3, IDO, and TGF-β. A secondary peroxidase-labeled polymer conjugated to goat anti-mouse immunoglobulins was applied for 60 min. The sections were developed with 3,3-diaminobenzidine (DAB) and counterstained with Mayer’s hematoxylin. Each slide was dehydrated using a graded series of alcohol concentrations and covered with a coverslip. The presence of positive cells on each slide was determined by a pathologist in a blinded manner.

**Fig 1:**
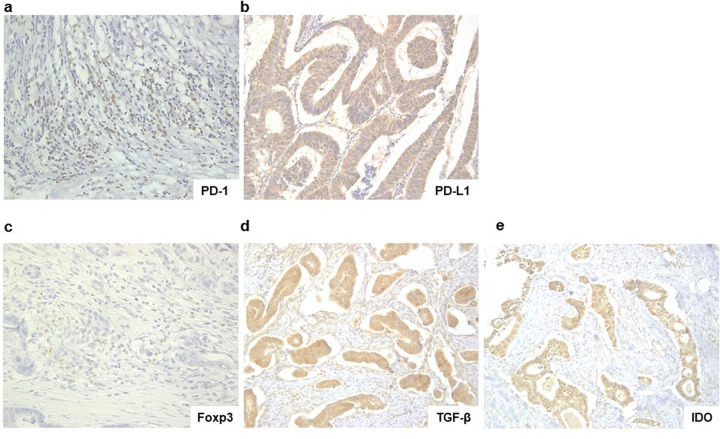
Immunohistochemistry. a. PD-1-positive expression in mononuclear cells in tumor tissue (x 400). b. PD-L1-positive expression in tumor cells (x 200). c. Foxp3-positive expression in mononuclear cells in tumor tissue (x 400). d. TGF-β-positive expression in tumor cells (x 200). e. IDO-positive expression in tumor cells (x 200).

As followed out previous experiment [7], PD-1 positivity was recorded when > 40% mononuclear cells in tumor tissue were stained in a ×400 high-power field of the tumor tissue section (Fig 1A). PD-L1 expression was predominantly located in the cytoplasm (Fig 1B). Staining intensity was scored as follows: 0, no staining; 1+, weak staining; 2+, moderate staining; and 3+, strong staining. Distribution was scored according to the percentage of PD-L1-positive cancer cells and then divided into quartiles as follows based on percentage staining: none, 0–5%; 1+, 6–25%; 2+, 26–50%; 3+, 51–75%; and 4+, 76–100%. A total score of > 3+ was defined as PD-L1-positive expression in tumor cells [7, 8]. We recorded Foxp3 positivity by counting more than 10 Foxp3-stained mononuclear cells in tumor tissue under ×200 high-power field (Fig 1C) [9]. TGF-β expression was predominantly located in the cytoplasm. TGF-β positivity was recorded when > 10% of the tumor cells were stained in the tumor tissue section (Fig 1D) [10]. IDO expression was predominantly located in the cytoplasm. Staining intensity was scored as follows: 0, no staining, 1+, weak; 2+, moderate; and 3+, strong. Distribution was scored according to the percentage of IDO-positive cancer cells and then divided into quartiles based on staining as follows: none, 0, 0–9%; 1+, 10–50%; 2+, 51–80%; 3+, 51–80%; and 4+, 81–100%. A total score of > 4+ was defined as IDO-positive expression in tumor cells (Fig 1E) [11].

### Statistical analysis

All statistical analyses were performed using JMP 8.0.1 (SAS, Cary, NC, USA). Continuous variables were compared using the Mann–Whitney U test, and categorical data were compared using chi-square (λ^2^) test. Survival curves were calculated using the Kaplan–Meier method and compared using log-rank tests. The prognostic potentials of the parameters were analyzed using univariate analysis. Relative risk and 95% confidence intervals (CI) were estimated using the Cox proportional hazards model with stepwise forward selection. Statistical significance was defined as p<0.05.

## Results

### Patients and tumor characteristics

The characteristics of patients according to the tumor sidedness are shown in Table 1. There were 27 patients of right-sided CRC and 75 patients of left-sided CRC. No significant difference was found in the patient and tumor characteristics depending on the sidedness. In terms of tumor immunity, PD-L1 expression was positively correlated with the sidedness of tumor. PD-L1 expression was higher in right-sided CRC than in left-sided CRC (62.9% vs. 30.6%, p<0.01). However, there was no significant difference in expression of PD-1, TGF-β, Foxp3, and IDO between the two groups.

**Table 1:** Patient and tumor characteristics.

Variables	Right-sided (n = 27)	Left-sided (n = 75)	p-value
**<Patient characteristics>**			
**Sex (male/female)**	**15/12**	**54/21**	**0.11**
**Age (year)**	**71±10**	**67±10**	**0.08**
**Adjuvant chemotherapy (-/+)**	**13:14**	**37:38**	**0.91**
**<Tumor characteristics>**			
**T stage (T1,2/T3,4)**	**9:18**	**34:41**	**0.27**
**Lymph node metastasis (-/+)**	**14:13**	**37:38**	**0.82**
**Primary tumor size (cm)**	**5.1±2.6**	**4.5±2.0**	**0.25**
**Tumor differentiation (tub1,2/muc, por)**	**5:22**	**6:69**	**0.13**
**Lymphatic invasion (-/+)**	**15:12**	**48:27**	**0.43**
**Venous invasion (-/+)**	**13:14**	**36:39**	**0.98**
**<Immune molecules>**			
**PD-1 (-/+)**	**19:8**	**54:21**	**0.87**
**PD-L1 (-/+)**	**10:17**	**52:23**	**0.003**
**TGF-β (-/+)**	**9:18**	**34:41**	**0.27**
**Foxp3 (-/+)**	**9:18**	**32:43**	**0.39**
**IDO (-/+)**	**19:8**	**52:23**	**0.92**

### Prognostic factors for CRC

In this study, univariate analysis identified expression of PD-1 (p = 0.02), PD-L1 (p = 0.01), and Foxp3 (p = 0.04) as significant prognostic factors for overall survival (OS) (Table 2). The OS rates was significantly poorer in the PD-1-positive group than in the PD-1-negative group (5-year OS rate, 78.7% vs. 93.6%, respectively). Furthermore, the PD-L1 and Foxp3 positive group showed poor survival compared to those of negative group (5-year OS rate, PD-L1: 76.3% vs. 93.0%, p = 0.01, Foxp3: 83.8% vs. 97.1%, p = 0.04, respectively).

**Table 2:** Univariate analysis of prognostic factors for colorectal cancer (CRC).

Variables	5-year survival rate (%)	p-value
**<OS>**		
**Age (<65 years/≥65 years)**	**89.6/89.0**	**0.95**
**Sex (male/female)**	**93.1/81.1**	**0.05**
**Tumor differentiation (tub1,2/muc, por)**	**89.4/87.5**	**0.83**
**T stage (T1,2/T3,4)**	**83.3/89.7**	**0.13**
**Lymph node metastasis (-/+)**	**93.9/83.9**	**0.14**
**Lymphatic invasion (-/+)**	**91.4/86.0**	**0.28**
**Venous invasion (-/+)**	**85.0/89.6**	**0.99**
**PD-1 (-/+)**	**93.6/78.7**	**0.02**
**PD-L1 (-/+)**	**93.0/76.3**	**0.01**
**TGF-β (-/+)**	**89.7/88.9**	**0.84**
**Foxp3 (-/+)**	**97.1/83.8**	**0.04**
**IDO (-/+)**	**90.4/86.8**	**0.25**
**Adjuvant chemotherapy (-/+)**	**89.2/89.2**	**0.67**
**Tumor location (right/left)**	**91.7/88.7**	**0.58**
**<DFS>**		
**Age (<65 years/≥65 years)**	**78.4/77.8**	**0.64**
**Sex (male/female)**	**80.7/72.7**	**0.29**
**Tumor differentiation (tub1,2/muc, por)**	**76.7/90.9**	**0.32**
**T stage (T1,2/T3,4)**	**71.4/78.7**	**0.48**
**Lymph node metastasis (-/+)**	**90.8/65.3**	**0.001**
**Lymphatic invasion (-/+)**	**86.4/64.1**	**0.003**
**Venous invasion (-/+)**	**84.2/72.7**	**0.18**
**PD-1 (-/+)**	**77.7/79.1**	**0.93**
**PD-L1 (-/+)**	**81.7/66.8**	**0.03**
**TGF-β (-/+)**	**80.4/76.4**	**0.72**
**Foxp3 (-/+)**	**87.2/71.9**	**0.04**
**IDO (-/+)**	**76.6/72.0**	**0.14**
**Adjuvant chemotherapy (-/+)**	**82.0/69.8**	**0.21**
**Tumor location (right/left)**	**84.8/76.2**	**0.43**

Regarding the disease-free survival (DFS), univariate analysis identified N (p = 0.001), lymphatic invasion (ly, p = 0.003), PD-L1 expression (p = 0.03), and Foxp3 expression (P = 0.04) as significant prognostic factors (Table 2). The PD-L1 and Foxp3 positive group showed poor survival compared to those of negative group (5-year DFS rate, PD-L1: 66.8% vs. 81.7%, p = 0.04, Foxp3: 66.8% vs. 81.7%, p = 0.03, respectively).

Multivariate analysis confirmed N (p = 0.01) as an independent risk factor for recurrence (relative risks of 3.39, Table 3).

**Table 3:** Multivariate analysis of prognostic factors in colorectal cancer (CRC).

Variables	Relative risk	95% CI	p-value
**CRC**			
**<OS>**			
**PD-1 +**	**1.70**	**0.51–5.59**	**0.38**
**PD-L1 +**	**2.88**	**0.89–9.29**	**0.08**
**Foxp3 +**	**2.88**	**0.58–14.3**	**0.15**
**<DFS>**			
**Lymph node metastasis +**	**3.39**	**1.20–9.57**	**0.01**
**Lymphatic invasion +**	**2.28**	**0.92–5.65**	**0.06**
**PD-L1**	**1.38**	**0.55–3.45**	**0.49**
**Foxp3 +**	**1.89**	**0.70–5.13**	**0.19**
**Right-sided**			
**<OS>**			
**IDO +**	**1.84**	**-**	**0.03**
**<DFS>**			
**Lymph node metastasis +**	**1.10**	**-**	**0.01**
**Venous invasion +**	**8.70**	**-**	**0.01**
**Left-sided**			
**<OS>**			
**PD-1 +**	**1.82**	**0.48–6.82**	**0.37**
**PD-L1 +**	**2.10**	**0.52–8.42**	**0.29**
**Foxp3 +**	**2.49**	**0.46–13.6**	**0.26**
**<DFS>**			
**Lymph node metastasis +**	**1.79**	**-**	**0.02**
**Lymphatic invasion +**	**3.13**	**0.32–30.5**	**0.28**
**PD-L1 +**	**1.43**	**0.19–10.5**	**0.72**

### Prognostic factors for right-sided CRC

**Fig 2:**
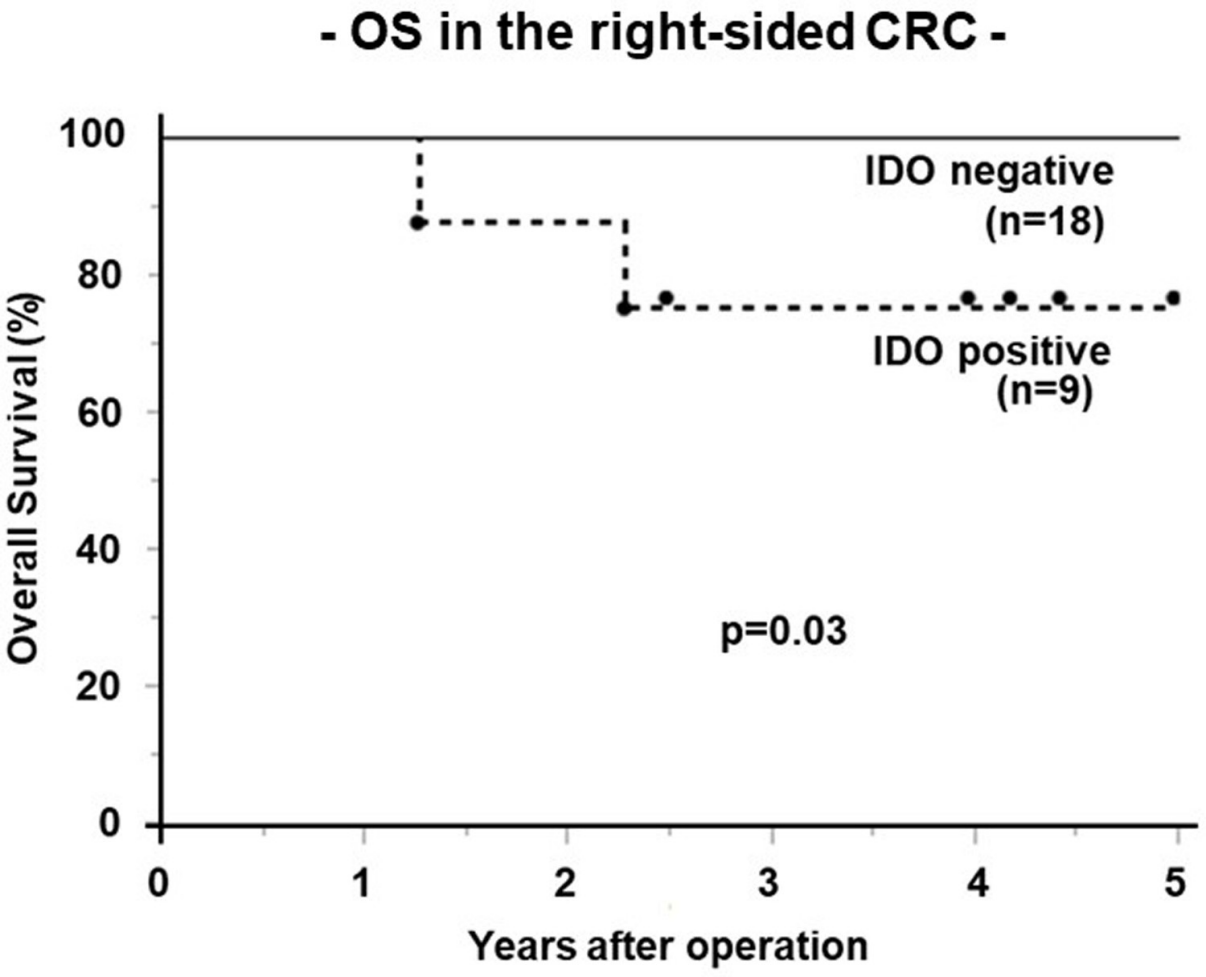
The overall survival rates in the right sided CRC. Kaplan–Meier analysis of 5-year overall survival for IDO expression. The OS rates was significantly poorer in the IDO-positive group (n = 9) than in the IDO-negative group (n = 18) (5-year OS rate, 75.0% vs. 100%, respectively).

In the right-side evaluation, univariate analysis identified only IDO expression (p = 0.03, Fig 2) as a significant prognostic factor for OS (Table 4). The OS rates was significantly poorer in the IDO-positive group than in the IDO-negative group (5-year OS rate, 75.0% vs. 100%, respectively). The PD-1, PD-L1, TGF-β and Foxp3 expression was not prognostic in right-sided CRC. Multivariate analysis confirmed IDO expression as independent prognostic indicators (relative risks of 1.84, Table 3).

**Table 4:** Univariate analysis of prognostic factors for right-sided colorectal cancer (CRC).

Variables	5-year survival rate (%)	P-value
**<OS >**		
**Age (<65 years/≥65 years)**	**100/88.1**	**0.34**
**Sex (male/female)**	**100/82.5**	**0.12**
**Tumor differentiation (tub1,2/muc, por)**	**89.9/100**	**0.49**
**T stage (T1,2/T3,4)**	**100/91.4**	**0.76**
**Lymph node metastasis (-/+)**	**100/82.1**	**0.12**
**Lymphatic invasion (-/+)**	**100/80.8**	**0.09**
**Venous invasion (-/+)**	**100/83.9**	**0.15**
**PD-1 (-/+)**	**93.3/87.5**	**0.56**
**PD-L1 (-/+)**	**100/78.7**	**0.06**
**TGF-β (-/+)**	**87.5/94.1**	**0.66**
**Foxp3 (-/+)**	**100/87.3**	**0.29**
**IDO (-/+)**	**100/75.0**	**0.03**
**Adjuvant chemotherapy (-/+)**	**91.6/91.6**	**0.90**
**<DFS>**		
**Age (<65 years/≥65 years)**	**87.5/83.5**	**0.79**
**Sex (male/female)**	**93.3/75.0**	**0.19**
**Tumor differentiation (tub1,2/muc, por)**	**100/81.5**	**0.33**
**T stage (T1,2/T3,4)**	**100/84.2**	**0.68**
**Lymph node metastasis (-/+)**	**100/68.4**	**0.02**
**Lymphatic invasion (-/+)**	**93.3/72.9**	**0.16**
**Venous invasion (-/+)**	**100/70.7**	**0.04**
**PD-1 (-/+)**	**89.5/75.0**	**0.35**
**PD-L1 (-/+)**	**87.8/80.0**	**0.49**
**TGF-β (-/+)**	**88.9/82.5**	**0.70**
**Foxp3 (-/+)**	**100/76.9**	**0.13**
**IDO (-/+)**	**88.8/75.0**	**0.29**
**Adjuvant chemotherapy (-/+)**	**92.3/77.9**	**0.35**

Regarding the DFS, univariate analysis identified N (p = 0.02) and v (p = 0.04) as significant prognostic factors (Table 4). None of immune marker was prognostic in right-sided CRC.

Multivariate analysis confirmed N (p = 0.01) and v (p = 0.01) as independent risk factors for recurrence (relative risks of 1.1 and 8.7, respectively, Table 3).

### Prognostic factors for left-sided CRC

**Fig 3:**
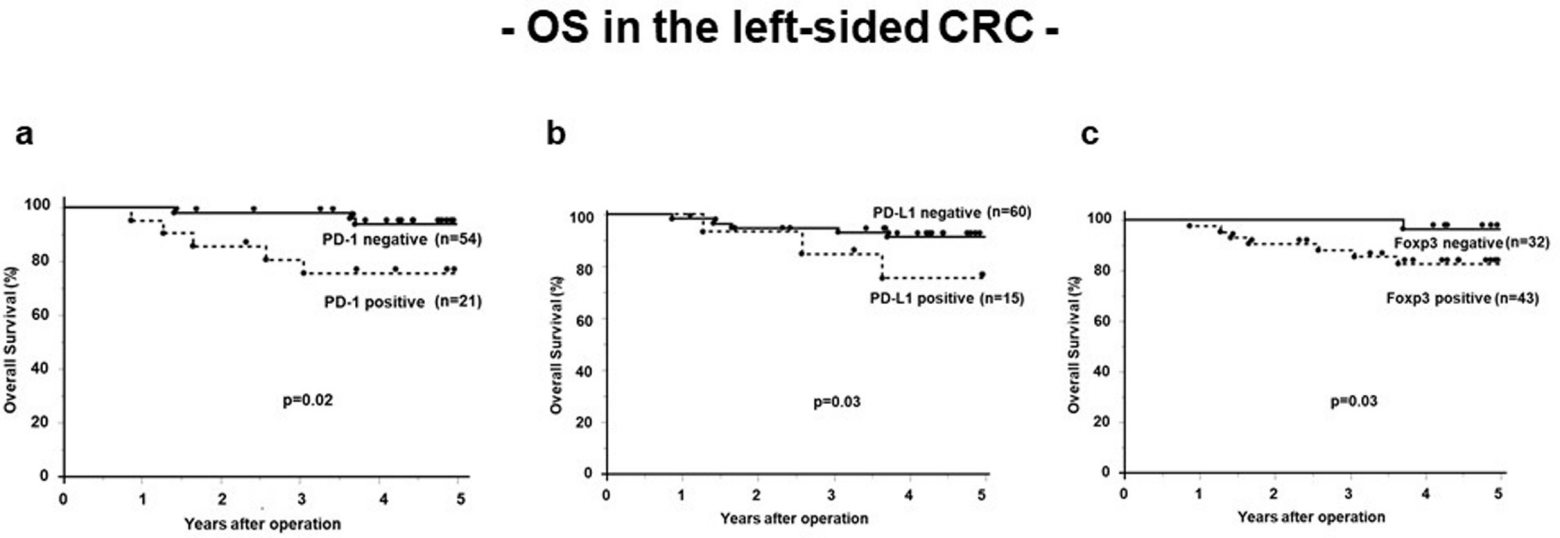
The overall survival rates in the left sided CRC. a. Kaplan–Meier analysis of 5-year overall survival for PD-1expression. The OS rates was significantly poorer in the PD-1-positive group (n = 21) than in the PD-1-negative group (n = 54) (5-year OS rate, 80.7% vs. 98.1%, respectively). b. Kaplan–Meier analysis of 5-year overall survival for PD-L1 expression. The OS rates was significantly poorer in the PD-L1-positive group (n = 15) than in the PD-L1-negative group (n = 60) (5-year OS rate, 75.4% vs. 91.4%, respectively). c. Kaplan–Meier analysis of 5-year overall survival for Foxp3 expression. The OS rates was significantly poorer in the Foxp3-positive group (n = 43) than in the Foxp3-negative group (n = 32) (5-year OS rate, 82.7% vs. 96.5%, respectively).

In the left side evaluation, univariate analysis identified PD-1 expression (p = 0.02), PD-L1 expression (p = 0.03) and Foxp3 expression (p = 0.03) as significant prognostic factors for OS (Table 5) in left-sided CRC. The OS rates was significantly poorer in the PD-1, PD-L1 and Foxp3 positive group than in the negative group (5-year OS rate, PD-1: 80.7% vs. 98.1%, PD-L1: 75.4% vs. 91.4%, Foxp3: 82.7% vs. 96.5%, respectively, Fig 3A–3C).

**Table 5:** Univariate analysis of prognostic factors for left-sided colorectal cancer (CRC).

Variables	5-year survival rate (%)	p-value
**<OS>**		
**Age (<65 years/≥65 years)**	**87.8/89.8**	**0.87**
**Sex (male/female)**	**91.8/80.4**	**0.10**
**Tumor differentiation (tub1,2/muc, por)**	**89.4/83.3**	**0.86**
**T stage (T1,2/T3,4)**	**80.0/89.3**	**0.07**
**Lymph node metastasis (-/+)**	**91.9/84.8**	**0.38**
**Lymphatic invasion (-/+)**	**89.2/88.2**	**0.66**
**Venous invasion (-/+)**	**85.7/91.6**	**0.51**
**PD-1 (-/+)**	**93.8/75.6**	**0.02**
**PD-L1 (-/+)**	**91.4/75.4**	**0.03**
**TGF-β (-/+)**	**90.6/87.1**	**0.58**
**Foxp3 (-/+)**	**96.5/82.7**	**0.03**
**IDO (-/+)**	**87.8/91.3**	**0.84**
**Adjuvant chemotherapy (-/+)**	**88.8/88.4**	**0.58**
**<DFS>**		
**Age (<65 years/≥65 years)**	**77.1/74.8**	**0.53**
**Sex (male/female)**	**78.2/71.4**	**0.49**
**Tumor differentiation (tub1,2/muc, por)**	**75.5/83.3**	**0.64**
**T stage (T1,2/T3,4)**	**66.6/77.1**	**0.36**
**Lymph node metastasis (-/+)**	**87.7/64.8**	**0.02**
**Lymphatic invasion (-/+)**	**84.6/60.8**	**0.007**
**Venous invasion (-/+)**	**78.9/73.7**	**0.67**
**PD-1 (-/+)**	**74.5/80.9**	**0.59**
**PD-L1 (-/+)**	**80.4/59.2**	**0.04**
**TGF-β (-/+)**	**78.4/74.1**	**0.73**
**Foxp3 (-/+)**	**83.9/70.2**	**0.19**
**IDO (-/+)**	**79.9/68.0**	**0.25**
**Adjuvant chemotherapy (-/+)**	**78.9/73.1**	**0.33**

Regarding the DFS, univariate analysis identified N (p = 0.02), ly (p = 0.007) and PD-L1(p = 0.04) as significant prognostic factors (Table 5). The DFS rates was significantly poorer in the PD-L1-positive group than in the PD-L1-negative group (5-year DFS rate, 59.2% vs. 80.4%, respectively). N was identified as an independent prognostic factor for DFS in left-sided CRC.

## Discussion

In this study, we showed the characteristics of immune markers associated with the sidedness of CRC. In right-sided CRC, PD-L1 was highly expressed than it was in left-sided CRC. In right-sided CRC, IDO positive group had significantly poor OS than negative group. No immune markers were related to the DFS. In left-sided CRC, the PD-L1 positive group showed significantly poor OS and DFS. Furthermore, the PD-1- and Foxp3-positive groups exhibited a significantly poor OS. This is the first report to show the characteristics of several tumor immune markers depending on the sidedness of the tumor.

Right-sided CRC was previously reported to have a high malignant potential, poor differentiation, and advanced stage, resulting in a poorer prognosis than that of left-sided CRC [12]. These differences are possibly due to differences in genetics, life-style, and dietary habits [12]. Chromosomal instability is more often observed in left-sided colon cancer than in right-sided, whereas defective genes include adenomatous polyposis coli (APC) and KRAS deletion [2]. In contrast, MSI-high, CIMP-high, BRAF mutation, and tumor-infiltrating lymphocytes (TIL) are characteristic in right-sided CRC [1, 3]. Both MSI and TIL are associated with a better prognosis and lower recurrence in patients with curative resection [13, 14]. Numerous recent studies have shown that mismatch repair status predicts the benefit of immune checkpoint blockade [15] and PD-1 and PD-L1 expression have been reported as predictive markers for PD-1/PD-L1 blockade. Furthermore, the MSI status correlated with PD-L1 expression in CRC [16]. PD-L1 expression was significantly higher in MSI-H/TML-high primary tumors from both left- and right sided CRC. These findings might suggest, at least partly, that the mucosal immunology differed depending on the sidedness.

PD-1 and PD-L1 play important roles in the regulation of the immune system and the maintenance of peripheral tolerance through T cell activation and tolerance [17]. We previously reported PD-1 expression as a predictor of poor prognosis in stage II/III gastric cancer [7] and CRC [5]. In our experiments, PD-1 was positively correlated with expression of the transcription factor, Foxp3, which is widely known to be involved in the development and function of regulatory T-cells (Tregs) [18]. Tregs have shown significant correlations with cancer progression and metastasis by inhibiting the T cell response via membrane-bound TGF-β1 [19]. We also previously reported that IDO expression is associated with poor prognosis and immunotolerance through attenuation of Treg activation in stage III gastric cancer [20]. IDO is an intracellular enzyme that catabolizes the conversion of tryptophan into kynurenine and is expressed in various types of human tumors [21]. IDO is expressed by tumor cells and tumor-draining lymph nodes and causes growth arrest and apoptosis of cytotoxic T and NK cells [22]. In addition, IDO also induces host Tregs [23] and is correlated with decreased patient survival [20]. Furthermore, high IDO-expressing tumors showed significantly lower numbers of TIL than IDO non-expressing tumors did [19, 20]. This is the first report to show the prognostic impact of IDO expression depending on the sidedness. IDO is the only prognostic factor in right-sided CRC.

A previous study reported the correlation between PD-1/PD-L1 expression and the sidedness of tumors. Berntsson et al. reported immune cell-specific PD-L1 expression is prognostic factor for OS in right-sided and left-sided CRC but not in rectal cancer. These authors also reported immune cell-specific PD-1 expression as a prognostic factor for OS in right- but not left-sided colon and rectal cancer. In our study, PD-1 and PD-L1 expression were prognostic factors for left- but not right-sided CRC. One reason for this observation might be the specificity of the expressing cells. We examined the PD-1 expression in immune cells and PD-L1 expression in tumor cells. Another study investigated the cytotoxic and regulatory T cell expression in CRC [24]. High Foxp3 expression was an independent prognostic factor only in patients with rectal cancer, but there was no significant interaction between Foxp3 expression and sidedness. In our study, Foxp3 was an independent prognostic factor for OS in left-sided CRC. One possible explanation for this finding is our classification of sidedness (we included rectal cancer in the left-sided group).

There were some limitations in this study that are worth mentioning. First, since the study was retrospective based on data from one institute, there may be a potential risk selection bias. The sample size was small. Furthermore, this study only used one analysis method (IHC). The biomarker expression should be confirmed by determining mRNA levels and the quantification of the infiltration levels of immune cell types by Gene set enrichment assay (GSEA) should be added in prospective studies. Nevertheless, we revealed the different characteristics of expressed immune molecules in CRC according to the tumor sidedness. However, the detailed mechanism including MSI status should be investigated in future studies.

In conclusion, this study reports the first comparison of the prognostic relevance of several immune-related molecules according to sidedness of CRC tumors. While PD-L1 was highly expressed in right-sided CRC, PD-L1 was the independent prognostic factor for OS and DFS in left-sided CRC. Furthermore, IDO was the prognostic factor for OS in right-sided CRC and Foxp3 was the prognostic factor for OS in left-sided CRC. These findings indicated that tumor location may be an important factor to consider when assessing immune response and therapeutic decisions in CRC patients.
